# Neonatal mortality of live births with congenital diaphragmatic hernia in São Paulo State, Brazil: Failure of care?

**DOI:** 10.1590/1984-0462/2025/43/2024138

**Published:** 2025-01-20

**Authors:** Ana Sílvia Scavacini Marinonio, Milton Harumi Miyoshi, Daniela Testoni Costa Nobre, Adriana Sanudo, Kelsy Catherina Nema Areco, Mandira Daripa Kawakami, Rita de Cássia Xavier Balda, Tulio Konstantyner, Carina Nunes Vieira e Oliveira, Paulo Bandiera-Paiva, Rosa Maria Vieira de Freitas, Monica La Porte Teixeira, Bernadette Cunha Waldvogel, Carlos Roberto Veiga Kiffer, Maria Fernanda Branco de Almeida, Ruth Guinsburg

**Affiliations:** a Universidade Federal de São Paulo, Escola Paulista de Medicina, São Paulo, SP, Brazil.; b Fundação Sistema Estadual de Análise de Dados, São Paulo, SP, Brazil.

**Keywords:** Congenital diaphragmatic hernia, Neonatal mortality, Neonatal intensive care, Epidemiological studies, Spatial distribution, Population, Hérnia diafragmática congênita, Mortalidade neonatal, Terapia intensiva neonatal, Estudos epidemiológicos, Distribuição espacial da população

## Abstract

**Objective:**

The aim of this study was to analyze if the healthcare organization of perinatal care and availability of referral neonatal intensive care units (NICU) impacted congenital diaphragmatic hernia (CDH) neonatal mortality in the period 2004–2020. This study analyzed the spatial distribution of neonatal deaths of live births with CDH in São Paulo State, Brazil, and its association with NICU beds’ availability.

**Methods:**

Population-based study of all live births in São Paulo State from mothers residing in the same State, from 2004 to 2020. CDH definition was based on WHO-ICD-10 codes; CDH-associated neonatal death was defined as death up to 27 days after birth of infants with CDH. The distribution of CDH-associated neonatal mortality (per 10,000 live births) and NICU beds’ availability (≥1 or not available) was mapped, and their association was evaluated by the Mann–Whitney test.

**Results:**

Among 10,246,686 live births, there were 1378 CDH-associated neonatal deaths across 124/645 (19.2%) municipalities of the State. The median CDH-associated neonatal mortality rate in municipalities with NICU beds was 1.22 (95%CI 0.99–1.51), similar to that found in municipalities with no NICU beds (1.40; 95%CI 1.15–1.67; p=0.224).

**Conclusions:**

CDH-associated neonatal deaths were spread throughout São Paulo State with no difference in CDH-associated neonatal mortality rates between municipalities with and without NICU beds available. These findings suggest the necessity of implementing regionalization strategies for CDH perinatal care in the State.

## RESUMO

**Objetivo:**

Analisar se a organização do cuidado perinatal e a disponibilidade de leitos de unidades de terapia intensiva neonatal (UTIN) no Estado de São Paulo influenciaram a mortalidade de nascidos vivos com hérnia diafragmática congênita (HDC) entre 2004 e 2020. Analisar a distribuição espacial dos óbitos neonatais de nascidos vivos com HDC no Estado de São Paulo e sua associação com a disponibilidade de leitos de UTIN.

**Métodos:**

Estudo de base populacional que incluiu todos os nascidos vivos do estado de São Paulo, filhos de mães residentes no mesmo estado em 2004–2020. A definição de HDC foi baseada nos códigos da 10^a^ Revisão da Classificação Internacional de Doenças da Organização Mundial da Saúde. Óbito neonatal associado à HDC foi definido como óbito até 27 dias após o nascimento de recém-nascidos com HDC. A distribuição da mortalidade neonatal associada à HDC (taxa por 10 mil nascidos vivos) e a disponibilidade de leitos de UTIN (≥1 ou não disponível) foi mapeada e sua associação avaliada por Mann-Whitney.

**Resultados:**

Entre 10.246.686 nascidos vivos, ocorreram 1.378 óbitos neonatais associados à HDC em 124/645 (19,2%) municípios do estado. A mediana da taxa de mortalidade neonatal associada à HDC nos municípios com leitos de UTIN foi de 1,22 (intervalo de confiança de 95% — IC95% 0,99–1,51), semelhante à encontrada nos municípios sem leitos de UTIN (1,40; IC95% 1,15–1,67; p=0,224).

**Conclusões:**

Os óbitos neonatais associados à HDC ocorreram de forma aleatória pelo estado de São Paulo, sem diferença nas taxas de mortalidade neonatal associadas à HDC entre os municípios com e sem leitos de UTIN disponíveis. Esses resultados sugerem a necessidade de implementação de estratégias de regionalização da assistência perinatal da HDC no estado.

## INTRODUCTION

Congenital diaphragmatic hernia (CDH) has an estimated global prevalence of 1.4:10,000 live births and a high neonatal mortality.^1^ Worldwide, the CDH-related neonatal mortality rate is 0.87:10,000 live births, with survival rates varying from 3.4% in some Latin American centers to 70% in high-income countries.^1,2,3^ Improving the survival of live births with CDH has been a challenge, even in high-income countries.^4^ On one hand, the high mortality is a consequence of the disease severity; on the other hand, the variation of CDH mortality may be related to differences in healthcare organization, availability of referral neonatal intensive care units (NICU), and quality of perinatal care.^3,5^ The lower mortality reported by high-income countries is associated with the antenatal diagnosis and maternal referral to centers experienced in CDH care (>6 CDH cases per year), trained multidisciplinary and surgical teams, and the presence of CDH management protocols covering the perinatal period.^6,7,8,9,10^ In many middle-income countries, the healthcare organization is deficient, and specialized neonatal units trained in CDH management are scarce.^5,11^

São Paulo State is the richest Brazilian State, with 645 municipalities and around 44 million inhabitants.^12^ São Paulo State has the second highest Human Development Index in the country (HDI 0.806 in 2021) and accounts for 32% of the national income.^13^ Despite these good economic indicators, the state has social and economic inequalities.^12^ In the State, some populational studies showed CDH prevalence of 1.58–1.67:10,000 live births, and mortality rates varying from 1.32:10,000 live births (2004–2015) to 1.58:10,000 live births (2006–2017);^14,15^ however, there are no studies providing information about the geographical distribution of CDH deaths in the State, which could guide public health policies.

In this context, the hypothesis of this study is that CDH-associated neonatal mortality is higher in municipalities without NICU beds. Therefore, our objective was to analyze the spatial distribution of deaths with CDH in São Paulo State, Brazil, from 2004 to 2020, and its association with NICU beds’ availability.

## METHOD

This is a population-based study of all live births from mothers residing in São Paulo State, between 2004 and 2020. Municipality of birth was considered a reference for this study. Thus, live births with a birth municipality outside São Paulo State or an unknown municipality of birth were excluded.

In São Paulo State, live birth and death certificates are completed by trained physicians and forwarded to the health offices and registry offices, which then send the information to Fundação Sistema Estadual de Análise de Dados (SEADE Foundation).^16^ For hospitalized live births, the live birth certificate is only released to the registry offices once the antenatal or admission diagnosis is confirmed. The SEADE Foundation makes the deterministic linkage from death with live birth records in order to identify birth information from all infants who died within 365 days after birth.^17^ For the study, SEADE Foundation provided two databases in which the municipality of birth was the smallest unit of location available: one related to all live births and the other related to all neonatal deaths in the State linked to the birth records. Both datasets were organized into a final database as described by Areco et al.^18^ The study was approved by the Ethics Committee of Universidade Federal de São Paulo, under the number 2.580.929, with waived informed consent.

CDH definition was based on the occurrence of the following World Health Organization ICD-10 codes in any line of death and/or live birth certificates: Q79.0 — congenital diaphragmatic hernia, K44 — diaphragmatic hernia, K44.0 — diaphragmatic hernia with obstruction, without gangrene, K44.1 — diaphragmatic hernia with gangrene, K44.9 — diaphragmatic hernia without obstruction or gangrene.^19^ CDH-associated neonatal death was defined as any infant death with CDH that occurred between 0 and 27 days after birth.

The CDH-associated neonatal mortality was calculated by dividing the number of CDH-associated neonatal deaths per 10,000 live births per municipality of birth and was presented as median and 95% confidence interval (CI).

The number of neonatal intensive care beds in each municipality was obtained by consulting the National Registry of Health Establishments (CNES).^20^ CNES registers the number of existing NICU beds (types I, II, or III) in each municipality of the country. The data reported in December of each year, starting from 2012, when the definitions for NICU beds were established,^21^ were used in this study.

The following demographic characteristics were described (in number and proportion) for all CDH-associated neonatal deaths: maternal age (<20, 20–34, and ≥35 years), maternal schooling (≤7, 8–11, and ≥12 years), parity (primiparous or multiparous), number of prenatal care visits (0, 1–6, and ≥7), single or multiple pregnancy, delivery mode (vaginal or cesarean section), gestational age (<28, 28–31, 32–36, 37–41, and ≥42 weeks), sex (male or female), first- and fifth-minute Apgar score (0–6 and ≥7), and isolated CDH or multiple malformations. Birthweight was described as mean and range.

Spatial distribution by quintiles of the CDH-associated neonatal mortality rate from 2004 to 2020 by the birthplace was shown by theme maps created for São Paulo State municipalities. Another thematic map was created to visually compare the birthplace of CDH-associated neonatal deaths and the availability of NICU beds, per municipality. In São Paulo State, the Regional Health Departments are responsible for the political administration and the distribution of health resources to the municipalities. Therefore, in both thematic maps, the limits of Regional Health Departments were highlighted. The availability of NICU beds was classified as available (≥1 bed) or not. The high number of municipalities without CDH-associated neonatal deaths in the state of São Paulo during the study period did not allow for any cluster analysis. Therefore, thematic maps were created to visualize the spatial distribution of municipalities with CDH-associated neonatal deaths and to visually compare the birthplace of CDH-associated neonatal deaths and the availability of NICU beds.

TerraView^®^ software version 5.5.0 (Instituto Nacional de Pesquisas Espaciais, São José dos Campos, Brazil) was used for the spatial distribution analysis.

Mann–Whitney test was applied to evaluate the association between the availability of NICU beds and the CDH-associated neonatal mortality rate. Finally, Kaplan-Meier estimator was used to identify the time of death according to the availability of NICU beds. Statistical significance was considered if p-value <0.05. Analytical procedures were performed using Stata 18^®^ (StataCorp LLC, Texas, USA).

## RESULTS

**Figure 1:**
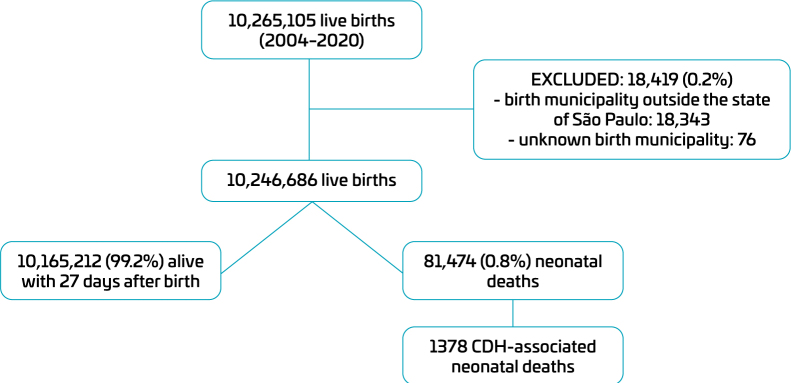
Flowchart of included infants, São Paulo State, Brazil, 2004–2020.

From 2004 to 2020, there were 10,265,105 live births in São Paulo State, Brazil. Among them, 10,246,686 were included in the study. Within this group, 1378 (0.01%) CDH-associated neonatal deaths were reported (Figure 1).

CDH: congenital diaphragmatic hernia.

Among CDH-associated neonatal deaths, maternal age was 20–34 years for 67.2% and schooling between 8 and 11 years for 54.5%. Most mothers were multiparous (59.3%), with a single pregnancy (97.4%); 68.4% had ≥7 prenatal care visits, and cesarean section occurred in 71.3%. Among the neonates that died with CDH, the birthweight was 2497 g (330–4610), 59.7% had gestational age between 37 and 41 weeks, 53.6% were male, and 39.0% had isolated CDH. Only 16.6% had first-minute Apgar ≥7, and 48.1% had fifth-minute Apgar ≥7 (Table 1).

**Table 1:** General characteristics of liveborn infants who died in the neonatal period with congenital diaphragmatic hernia, state of São Paulo, Brazil, 2004–2020.

	CDH-associated neonatal deaths
Maternal age (years) (%)	n=1378
<20	12.8
20–34	67.2
≥35	20.0
Maternal schooling (years) (%)	n=752
≤7	28.5
8–11	54.5
≥12	17.0
Primiparous (%)	n=796
Yes	40.7
Prenatal care visits (%)	n=1201
0	1.0
1–6	30.6
≥7	68.4
Pregnancy (%)	n=1378
Single	97.4
Delivery (%)	n=1352
Cesarean section	71.3
Gestational age (weeks) (%)	n=1218
<28	2.8%
28–31	7.3%
32–36	29.1%
37–41	59.7%
≥42	1.1%
Birthweight (grams)	n=1333
Mean (range)	2497 (330–4610)
Sex (%)	n=1377
Male	53.6
1st-minute Apgar score ≥7 (%)	n=741
	16.6
5th-minute Apgar score ≥7 (%)	n=743
	48.1
CDH classification (%)	n=1378
Isolated	39.0

CDH: congenital diaphragmatic hernia; n: total number available for the variable.

For most CDH-associated neonatal deaths (908/1378; 65.9%), the municipality of birth was the same one reported for maternal residence. Most neonates that died with CDH (1293/1378; 93.8%) were born and died in the same municipality.

**Figure 2:**
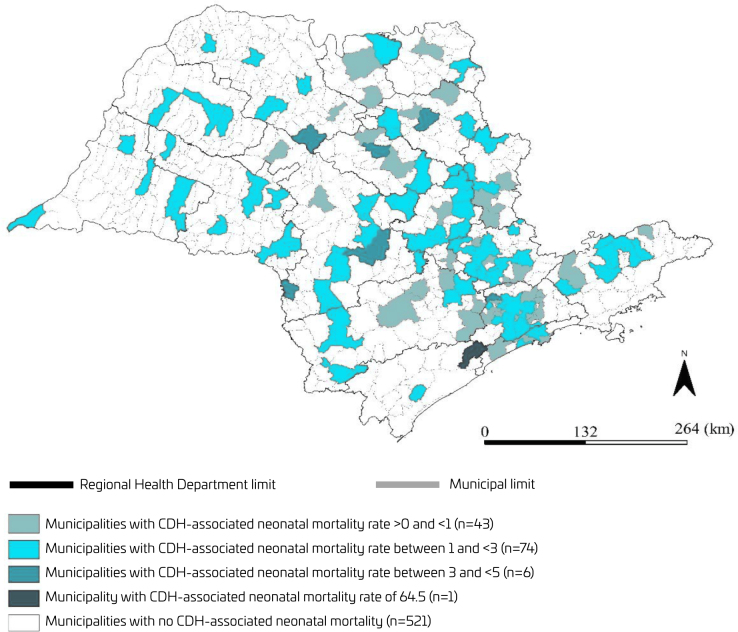
Distribution of congenital diaphragmatic hernia-associated neonatal mortality by municipality, in the 17-year study period (São Paulo State, Brazil, 2004–2020).

CDH: congenital diaphragmatic hernia; n: number of municipalities.

In the 17-year period, 124/645 (19.2%) municipalities in the State of São Paulo registered at least one CDH-associated neonatal death, with a CDH-associated neonatal mortality rate of 1.53 per 10,000 live births. The absolute number of CDH-associated neonatal deaths ranged from 0 to 558 among the São Paulo State municipalities. The municipality with 558 CDH-associated neonatal deaths was the capital of the state, São Paulo, followed by Campinas with 67 deaths and Ribeirão Preto with 61. The CDH-associated neonatal mortality rate of these three municipalities was, respectively, 1.74, 1.93, and 3.23 per 10,000 live births. These three municipalities (São Paulo, Campinas, and Ribeirão Preto) concentrated almost half of CDH-associated neonatal deaths (686/1378) that occurred in the State. CDH associated neonatal mortality ranged from 0 to 64.5 per 10,000 live births (median: 1.26; 95%CI 1.02–1.46). The higher CDH-associated neonatal mortality rate was found in one municipality with 155 live births between 2004 and 2020 and one CDH-associated neonatal death. CDH-associated neonatal deaths were observed in all Regional Health Departments (Figure 2).

**Figure 3:**
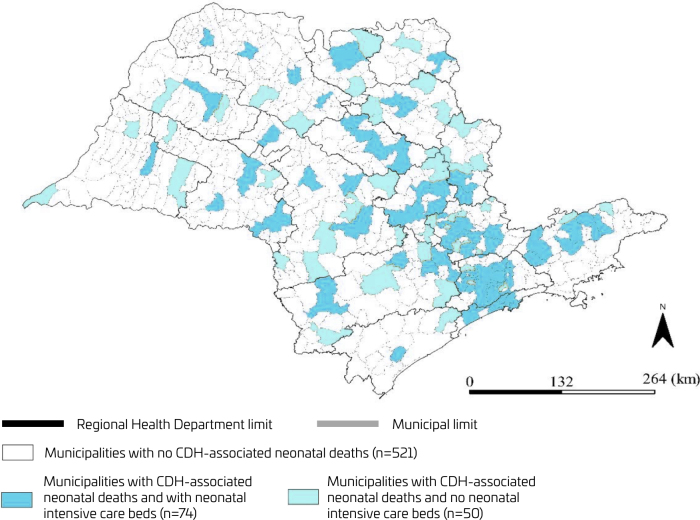
Distribution of municipalities with congenital diaphragmatic hernia-associated neonatal deaths and neonatal intensive care unit beds (São Paulo State, Brazil, 2004–2020).

CDH: congenital diaphragmatic hernia; n: number of municipalities.

**Figure 4:**
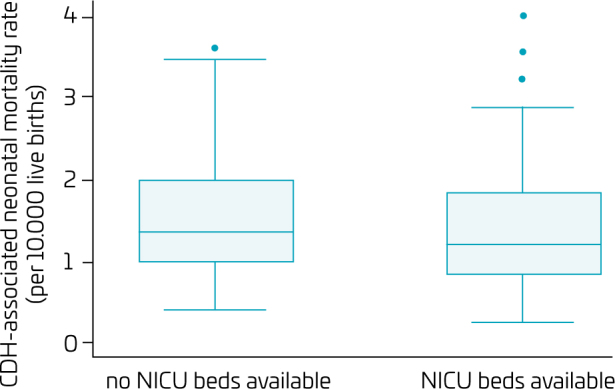
Boxplot of the congenital diaphragmatic hernia-associated neonatal mortality rate according to the availability of neonatal intensive care unit beds, by municipality (São Paulo State, Brazil, 2004–2020).

Among the 124 municipalities with CDH-associated neonatal deaths registered, 60% (74/124) NICU beds were available. With the exception of the Araraquara and Santos, all Regional Health Departments experienced CDH-associated neonatal deaths in municipalities without NICU beds (Figure 3). The median CDH-associated neonatal mortality rate was 1.22 (95%CI 0.99–1.51) in the 74 municipalities with available NICU beds. This rate was 1.40 (95%CI 1.15–1.67) in the 50 municipalities without available NICU beds (Mann–Whitney; p=0.224) (Figure 4).

The outlier for CDH-associated neonatal mortality rate (64.5 per 10,000 live births) was removed from the “no NICU beds availability” boxplot.

CDH: congenital diaphragmatic hernia; NICU: neonatal intensive care unit.

Kaplan-Meier analysis showed that the median time of CDH-associated neonatal deaths was 24 h after birth (P25-75: 6 to 80). For municipalities with CDH-associated neonatal deaths and available or non-available NICU beds, the median time of death was, respectively, 24 h (P25-75: 6 to 79) and 24 h (P25-75: 5 to 96).

## DISCUSSION

This population-based study, conducted in São Paulo State municipalities, Brazil, from 2004 to 2020, evaluated the CDH-associated neonatal mortality and the availability of neonatal intensive care. In the 17-year period, there were 1378 CDH-associated neonatal deaths distributed in 124/645 (19%) of the State’s municipalities. The median CDH-associated neonatal mortality rate in the State’s municipalities was 1.26 per 10,000 live births (95%CI 1.02–1.46). Neonatal intensive care beds were not available in 50/124 (40%) municipalities with CDH-associated neonatal deaths registered, and the median CDH-associated neonatal mortality rate in these municipalities was similar to the rate found in 74/124 (60%) municipalities with at least 1 NICU bed available.

Currently, despite the introduction of new therapeutic and supportive modalities to treat CDH, the optimal management is unknown.^22^ The higher survival rate has been attributed to better antenatal diagnosis, referral of deliveries to tertiary centers, and the presence of well-resourced intensive care units with organized, integrated, and experienced multidisciplinary teams.^5,23,24^ Despite challenges stemming from the complexity of care,^25^ standardization of treatment and elimination of disparities in clinical management contribute to a higher survival rate of live births with CDH.^26^

Brazil still lacks reliable and complete sources of essential information on CDH mortality that would provide subsidies for public policies involving CDH perinatal care.^15^ In our study, the high CDH-associated neonatal mortality rate and the wide distribution of CDH-associated neonatal deaths throughout São Paulo State’s municipalities, many of them occurring in municipalities without access to NICU beds, reveal a weak organization of CDH perinatal care in the state, with a lack of established referral centers and flows for pregnant women to be referred to them. Therefore, the choice of birthplace is not directed by perinatal expertise in CDH management, which is crucial for achieving better outcomes.^27^

The three municipalities that concentrate the highest number of CDH-associated neonatal deaths (São Paulo, Campinas, and Ribeirão Preto) are among the seven most populous municipalities and among the first six with the highest number of live births in the state.^12^ Furthermore, these municipalities have large referral centers for neonatal care and specialized teaching hospitals, with the availability of neonatal intensive care beds and professional expertise.^28^ Thus, even without public policies for CDH perinatal management in São Paulo State, centers considered as references in perinatal care in the State could have attracted a greater number of cases (and, consequently, of deaths) either by medical referral or by maternal choice, suggesting that there is a rudimentary informal referral flow for CDH births, although this has not been documented or confirmed.

Possibly, CDH live births in municipalities with no NICU beds died without adequate resources, before or during neonatal transport or even after reaching a tertiary center with specialized and surgical care.^29^ However, the present study also demonstrated that the availability of NICU beds in the municipality of CDH birth did not affect the CDH-associated neonatal mortality rate, which remained high. Therefore, the mere availability of beds is not sufficient to reduce CDH deaths. Hospital-based studies from high-income countries report better outcomes if the birth of infants with CDH occurs in centers with a volume of care higher than six per year, which may provide a more holistic and coordinated care for infants with this complex anomaly.^7,8,30^ In Texas, centers that handled 75 or more CDH cases between 2013 and 2021 had lower lethality rates compared to centers that handled up to 20 cases during the same period (18% vs. 27%), despite the higher volume in centers admitting more severe cases.^10^

In this context, process and structural factors should be taken into consideration for quality improvement policies.^31^ Considering that the regionalization of care, with the provision of quality prenatal counseling service, identification of a reference center with more experienced and organized teams improves the outcomes of live births with CDH.^5^ The establishment of public policies for CDH management, with regionalization of perinatal care and designation of specific obstetric and neonatal units equipped and able to offer a state-of-the-art care for CDH infants before, during, and after birth in São Paulo State are necessary to modify the scenario observed in this study.

This study has several limitations. The database was provided by SEADE Foundation after linkage and anonymization, a process that relies on a time-consuming manual component; therefore, the most recent year available for the study was 2020. The database originates from live birth and death certificates, and there is a risk of information bias since it depends on diagnoses notification. The database does not include information on birth hospitals, maternal diseases, procedures during pregnancy, CDH characteristics, and management, which makes it difficult to discuss differences in performance between municipalities. Regarding NICU beds in the State, CNES provides monthly data on the number of NICU beds, which is subject to variation over time. The classification of NICU beds into types I, II, and III has been available since 2012.^21^ Therefore, in order to assess the availability of NICU beds per municipality, we considered the period of 2012–2020, which may overestimate the data during the initial period of the study. For comparison between mortality and the availability of neonatal ICU beds, the municipality was the smallest area available for analysis. Due to the lack of information about the hospital where the birth occurred, we could not assert that a CDH birth occurred in a municipality with available NICU beds and took place in a hospital with NICU beds. Finally, the high number of municipalities without CDH-associated neonatal deaths in the São Paulo State over the years studied does not allow to perform any cluster analysis, thus, only a descriptive analysis was done. Despite these limitations, this study is one of the first population-based evaluations of the spatial distribution of CDH live births and CDH mortality in one of the richest and most populous regions of a middle-income country.

During a 17-year period, this study has shown a wide distribution of CDH-associated neonatal deaths throughout São Paulo State municipalities, with no difference between municipalities with or without available NICU beds. These findings underscore the necessity of adopting regionalization strategies for CDH perinatal care within the state.

## Data Availability

The declare that originated the article is available with the corresponding author. CAAE: 86014518.9.0000.5505

## References

[1] Politis MD, Bermejo-Sánchez E, Canfield MA, Contiero P, Cragan JD, Dastgiri S (2021). Prevalence and mortality in children with congenital diaphragmatic hernia: a multicountry study. Ann Epidemiol.

[2] Paoletti M, Raffler G, Gaffi MS, Antounians L, Lauriti G, Zani A (2020). Prevalence and risk factors for congenital diaphragmatic hernia: a global view. J Pediatr Surg.

[3] Cruz-Martínez R, Etchegaray A, Molina-Giraldo S, Nieto-Castro B, Guevara EG, Bustillos J (2019). A multicentre study to predict neonatal survival according to lung-to-head ratio and liver herniation in fetuses with left congenital diaphragmatic hernia (CDH): hidden mortality from the Latin American CDH Study Group Registry. Prenat Diagn.

[4] Gupta VS, Harting MT, Lally PA, Miller CC, Hirschl RB, Davis CF (2023). Mortality in congenital diaphragmatic hernia: a multicenter registry study of over 5000 patients over 25 years. Ann Surg.

[5] Lum LC, Ramanujam TM, Yik YI, Lee ML, Chuah SL, Breen E (2022). Outcomes of neonatal congenital diaphragmatic hernia in a non-ECMO center in a middle-income country: a retrospective cohort study. BMC Pediatr.

[6] Apfeld JC, Kastenberg ZJ, Gibbons AT, Carmichael SL, Lee HC, Sylvester KG (2020). Treating center volume and congenital diaphragmatic hernia outcomes in California. J Pediatr.

[7] Bucher BT, Guth RM, Saito JM, Najaf T, Warner BW (2010). Impact of hospital volume on in-hospital mortality of infants undergoing repair of congenital diaphragmatic hernia. Ann Surg.

[8] Grushka JR, Laberge JM, Puligandla P, Skarsgard ED (2009). Canadian Pediatric Surgery Network Effect of hospital case volume on outcome in congenital diaphragmatic hernia: the experience of the Canadian Pediatric Surgery Network. J Pediatr Surg.

[9] Masahata K, Usui N, Shimizu Y, Takeuchi M, Sasahara J, Mochizuki N (2020). Clinical outcomes and protocol for the management of isolated congenital diaphragmatic hernia based on our prenatal risk stratification system. J Pediatr Surg.

[10] Peiffer SE, Mehl SC, Powell P, Lee TC, Keswani SG, King A (2024). Treatment facility case volume and disparities in outcomes of congenital diaphragmatic hernia cases. J Pediatr Surg.

[11] Global PaedSurg Research Collaboration (2021). Mortality from gastrointestinal congenital anomalies at 264 hospitals in 74 low-income, middle-income, and high-income countries: a multicentre, international, prospective cohort study. Lancet.

[12] Instituto Brasileiro de Geografia e Estatística [homepage on the Internet] Cidades e Estados.

[13] Atlas Brasil [homepage on the Internet] (2021). Ranking.

[14] Marinonio AS, Miyoshi MH, Costa-Nobre DT, Sanudo A, Areco KC, Kawakami MD (2023). Congenital diaphragmatic hernia in a middle- income country: persistent high lethality during a 12-year period. PLoS One.

[15] Maia VO, Pavarino E, Guidio LT, Souza JP, Ruano R, Schmidt AF (2022). Crossing birth and mortality data as a clue for prevalence of congenital diaphragmatic hernia in Sao Paulo State: a cross sectional study. Lancet Reg Health Am.

[16] Brazil Ministério da Saúde. Secretaria de Vigilância em Saúde [homepage on the Internet]. Portaria n^o^ 116, de 11 de fevereiro de 2009. Regulamenta a coleta de dados, fluxo e periodicidade de envio das informações sobre óbitos e nascidos vivos para os Sistemas de Informações em Saúde sob gestão da Secretaria de Vigilância em Saúde. [cited 2023 Jan 05].

[17] Waldvogel BC, Morais LC, Perdigão ML, Teixeira ML, Freitas RM, Aranha VJ (2019). Experiência da Fundação Seade com a aplicação da metodologia de vinculação determinística de bases de dados. Ensaio & Conjuntura.

[18] Areco KN, Konstantyner T, Bandiera-Paiva P, Balda RC, Costa-Nobre DT, Sanudo A (2021). Operational challenges in the use of structured secondary data for health research.. Front Public Health.

[19] World Health Organization (2004). ICD-10: International statistical classification of diseases and related health problems, tenth revision..

[20] Brazil. Ministério da Saúde Secretaria de Atenção à Saúde. Cadastro Nacional dos Estabelecimentos de Saúde. Departamento de Informática do Sistema Único de Saúde [homepage on the Internet].

[21] Brazil. Ministério da Saúde (2012). Gabinete do Ministro. Portaria n^o^ 930, de 10 de maio de 2012. Define as diretrizes e objetivos para a organização da atenção integral e humanizada ao recém-nascido grave ou potencialmente grave e os critérios de classificação e habilitação de leitos de Unidade Neonatal no âmbito do Sistema Único de Saúde (SUS).

[22] Pala C, Blake SM (2023). One size does not fit all: congenital diaphragmatic hernia management in neonates. Neonatal Netw.

[23] Hayakawa M, Ito M, Hattori T, Kanamori Y, Okuyama H, Inamura N (2013). Effect of hospital volume on the mortality of congenital diaphragmatic hernia in Japan. Pediatr Int.

[24] Puligandla P, Skarsgard E, Baird R, Guadagno E, Dimmer A, Ganescu O (2024). Diagnosis and management of congenital diaphragmatic hernia: a 2023 update from the Canadian Congenital Diaphragmatic Hernia Collaborative. Arch Dis Child Fetal Neonatal Ed.

[25] Dimmer A, Baird R, Puligandla P (2024). Role of practice standardization in outcome optimization for CDH. World J Pediatr Surg.

[26] Ito M, Terui K, Nagata K, Yamoto M, Shiraishi M, Okuyama H (2021). Clinical guidelines for the treatment of congenital diaphragmatic hernia. Pediatr Int.

[27] Maldonado BN, Lanoue J, Allin B, Hargreaves D, Knight M, Gale C (2024). Place of birth and postnatal transfers in infants with congenital diaphragmatic hernia in England and Wales: a descriptive observational cohort study. Arch Dis Child Fetal Neonatal Ed.

[28] Barata LR, Bittar OJ, Magalhães A, Alves SA, Carvalho ER (2009). Comparação de grupos hospitalares no Estado de São Paulo. Rev Adm Saúde.

[29] Balayla J, Abenhaim HA (2014). Incidence, predictors and outcomes of congenital diaphragmatic hernia: a population-based study of 32 million births in the United States. J Matern Fetal Neonatal Med.

[30] Wang Y, Honeyford K, Aylin P, Bottle A, Giuliani S (2019). One-year outcomes for congenital diaphragmatic hernia. BJS Open.

[31] Morche J, Mathes T, Jacobs A, Pietsch B, Wessel L, Gruber S (2020). Relationship between volume and outcome for surgery on congenital diaphragmatic hernia: a systematic review. J Pediatr Surg.

